# Hidden Tales of Ebola: Airing the Forgotten Voices of Ugandan “Ebola Nurses”

**DOI:** 10.1177/10436596211017968

**Published:** 2021-06-06

**Authors:** Isaac Okello Wonyima, Susan Fowler-Kerry, Grace Nambozi, Charlotte Barry, Jeanie Wills, Yolanda Palmer-Clarke, Rozzano C. Locsin

**Affiliations:** 1 Uganda Martyrs University Uganda, Kampala, Uganda; 2 University of Saskatchewan, Saskatoon, Saskatchewan, Canada; 3 Mbarara University of Science and Technology, Mbarara, Uganda; 4 Florida Atlantic University, Boca Raton, FL, USA

**Keywords:** nursing practice, transcultural health, women’s health, phenomenology, Ebola

## Abstract

**Introduction:**

According to the Centers for Disease Control and Prevention, Ebola has affected the lives of thousands, including health care workers. With few studies describing the experience of nurses who survived Ebola, the study aimed to describe Ugandan nurses’ experiences.

**Method:**

Using a phenomenological design, in-depth interviews were conducted among five Ugandan nurses who contracted Ebola and survived.

**Result:**

Thematic analysis revealed themes of expectations of dying, hopelessness, loneliness, and betrayal by family, community, and the health system.

**Discussion:**

Results support the need for policies targeting holistic practice protocols to protect all health care professionals during future outbreaks. Last, nursing survivors should have access to government-guaranteed support programs, including free health care and financial stipends. These results and recommendations transcend to the current reality of living with COVID-19 (coronavirus disease 2019). Efficient practice protocols could protect all rights and privileges and contribute to access to treatment and stigma removal.

## Introduction

Before the current coronavirus pandemic, many parts of the world suffered from outbreaks of varied viral illnesses. Ebola virus disease (EVD) is one such disease, which has over the years claimed the lives of thousands globally, especially citizens of developing countries. According to the Centers for Disease Control and Prevention (CDC), the outbreak between the years 2014 and 2016 was significant in the losses caused by the disease. During this time, there were 28,616 cases of EVD and 11,310 deaths reported in Guinea, Liberia, and Sierra Leone. This outbreak spread quickly and became a global epidemic (CDC, 2019). Uganda experienced two Ebola outbreaks. In 2000-2001 in Gulu where 425 cases were reported and 224 deaths, 22 were health care worker (Hoyos, 2000). In 2007, an outbreak occurred in Bundibugyo;14 health care workers were infected (Okware, 2015).

While the stories of survivors abound, there is a shortage in the literature about the Ugandan nurses who worked on the front line caring for Ebola patients, many of whom became infected. Sadly, not much research explores their experience living with the disease. This research explored the experiences of nurse survivors who contracted EVD while caring for infected parents. The purpose of the study was to describe the experiences of nurse survivors who contracted EVD while caring for infected patients. The research question was, “What was it like living through and surviving EVD?”

## Background

In the 1970s, the first EVD outbreak occurred in the Democratic Republic of the Congo in a village near the Ebola river; thus, the virus was called Ebola in memory of its origin. The second outbreak occurred in South Sudan in the same year. Although there were earlier outbreaks, West Africa saw its largest Ebola outbreak in 2014. Researchers estimated that the caseload was nearly 30,000 and over 11, 000 died (B. S. Hewlett, 2016; Hewlett & Amola, 2003; Marshall & Smith 2015; Sastry & Dutta, 2017). In 2019, Ebola again surfaced in Uganda and the Democratic Republic of the Congo when it was declared a public health emergency, and in 2020 was declared the largest second largest Ebola outbreak on record (World Health Organization [WHO], 2020, Lamunu et al. 2004).

Uganda experienced three outbreaks between 2000 and 2012, the largest and most destructive occurred in the Gulu region in 2000 (Okware, 2015; Okware et al. 2015). This outbreak occurred during the rainy season when malaria rates were highest, thus complicating the diagnosis. As the outbreak accelerated, health care workers were not alerted that the infectious agent was Ebola nor were they informed about the disease and the mode of transmission. Nurses, during this period, were working without personal protective equipment (PPE) and guidelines to implement precautions. As a result, 64% of health care workers became infected (Raven et al., 2018).

### Ebola Virus Disease

EVD is deadly and attacks the human body. This viral infection spreads by direct contact with bodily fluids of a person who is sick with or has died from EVD and causes hemorrhagic fevers. In its initial stages, EVD is hard to diagnose and exhibits symptoms common to malaria, influenza, or typhoid fever (CDC, 2014; Okware, 2015). EVD symptoms include fever, aches and pains, weakness and fatigue, gastrointestinal symptoms, including diarrhea and vomiting, abdominal (stomach) pain, unexplained hemorrhaging, bleeding, or bruising.

Contracting and surviving a deadly illness such as EVD is difficult to forget, even with time. The stories of survivors reveal many challenges such as stigma, rejection by their community and family members, loss of properties, the fear of communal burial by surviving strangers and not family members (Atwine, 2015; Bell et al., 2017; B. L. Hewlett & Hewlett, 2005; Kollie et al., 2017; Marshall & Smith, 2015; Matua, 2014; Schmidt-Sane et al., 2020; Wester & Giesecke, 2019; WHO, 2015). These experiences, in addition to the physical consequences of surviving, compromise the life-expectancy and quality of life of infected nurses (Hoyos, 2000; Kinsman, 2012; Okware et al., 2002). The lasting effects are particularly true for nurses who cared for patients and became infected with EVD (Cheung, 2015; Kamara, et al., 2017).

### Purpose and Research Question

This research sought to answer the research question: “What was it like being a nurse living through and surviving Ebola?” It therefore describes the experiences of Ugandan nurse survivors who cared for Ebola patients in Gulu, a city in the northern region of Uganda. Their stories of survival can inform preparations for future outbreaks and the development of interventions needed to undergird the prevention of EVD and other infectious diseases, support for those infected in the acute phase, and for well-being for those with long-term survival (Krouskos et al., 2017; O’Brien & Tolosa, 2016).

## Method and Design

The team employed a qualitative phenomenological design based on the work of van Manen to gain a deeper understanding of the experiences of five nurses who contracted EVD while caring for patients with the disease. As a qualitative approach, phenomenology allows for a “deeper understanding of the nature of the meaning of our everyday experiences” (p. 9). This approach to qualitative research is both descriptive and interpretive as it serves to offer description, interpretation, and critical self-reflection into the “world as world” (van Manen, 2006, p. 5). Thus, through this phenomenological study, the researchers were able to enter the “lifeworld” of participants while examining every element of their experience for interpretation of what the phenomenon meant for these nurses (van Manen, 2006). This method, essentially, allowed the investigators to reach in, examine, and give meaning to the lived experiences of select nurses who were on the front line during the Ugandan EVD crisis.

### Sample and Setting

Conducted in Gulu district, situated in northern Uganda, from May to September 2015, there were five participants in this study. Eight nurses fit the study criteria, but only five could be located and agreed to participate. They ranged in age from 30 to 60 years. They were all staff nurses who worked on hospital wards during the Ebola outbreak. The mean year of employment was 20 years, and the range was 16 to 31 years. All participants had at least 1 month of experience caring for patients with Ebola and 14 to 18 days of experience as patients with Ebola.

### Data Collection

The researchers purposively selected the first participant by examining hospital records. The first participant assisted the team to locate other survivors. The researchers contacted the referred participants and communicated the research purpose and process to them before requesting informed consent. Once participants consented, they agreed to meet and complete a semistructured face-to-face interview at a location of their choice.

Interviews lasted up to 1 hour but no more. English was the primary language used during interviews. However, several participants were more comfortable speaking Bantu. For these interviews, the researchers translated the questions to the local language for participants. The researchers recorded the interviews. To maintain confidentiality, participants were assigned numbers 1 to 5. Participants were asked to reconstruct their experiences during the period of working with EVD patients, contracting the disease, and living after they were declared healed. As the conversations ensued, probing questions were added to clarify and add information as was necessary. There were follow-up interviews that allowed participants to review their transcripts to clarify, add, or redact information.

### Analysis

When analyzing the data, the researchers used van Manen’s four stages of data analysis, which are (1) uncovering thematic aspects, (2) isolating thematic statements, (3) composing linguistic transformations, and (4) gleaning thematic descriptions. Congruent with van Manen’s approach, the researchers were also quick to bracket their feelings, emotions, and experiences to ensure that they were able to glean from the stories the essence of the experience of being a nurse living through an Ebola outbreak (van Manen, 2006). To stay true to the data and the phenomenological spirit, themes were named using the direct words of the speaker.

The team worked on transcribing data from audiotapes verbatim and as soon as possible after each interview and made the necessary edits and checks to ensure that participants’ views were correctly reflected. Analysis of the data included an iterative process of reading and rereading. Transcripts were analyzed multiple times, line by line individually and collectively by the research team to capture the essence of participants’ stories and to establish the common interpretations of the phenomenon. Thus, the researchers examined every word and every sentence to unearth the essence of the data. This method allowed the researchers to explore and reexamine data to identify words describing the participants’ lived experiences of surviving Ebola. From this process, 26 themes emerged initially, which on reflection and reevaluation (grouping and regrouping like themes), six overarching themes emerged.

### Ethical Considerations

Before setting out on the study, the investigators obtained permission to conduct this study from the Institutional Research and Ethics Committee of Lacor Hospital and District Health Officer, Gulu District, Uganda. Participants were free to withdraw from the study at any time if they felt uncomfortable continuing. A counselor was available to offer counseling services should the need arise. Throughout the process, one participant broke down, she was told she could discontinue the interview and or have access to the counseling services provided. The participant opted to continue without counseling.

### Study Limitations

The study was conducted primarily in English and analyzed using Western qualitative methods. Notedly, the best expression of sadness in Africa is rendered anecdotally, so drawing as a method and using local language and idioms could have provided a more detailed description of their lived experiences as some expressions have no direct English translations. The use of African philosophy, such as Ubuntu in times of such life-threatening illness, could have unearthed deeper meanings of the participants’ lived experiences.

## Findings

The five Ebola nurse survivors recalled and retold their stories as it happened. The first three of six themes described their perceptions as they struggled within the acute phase. These are described as if being experienced in the moment, such as living in constant fear of dying, feeling hopeless yet hopeful in God’s mercy, and longing to connect with others. Three other themes were contextualized in the past but continuing in the present. These themes include such as suffering health disorders, being ostracized, and feeling neglected and betrayed by organizational systems.

### Living in Constant Fear of Dying

This theme emerged from participants descriptions of their experience of contracting EVD while caring for infected patients. Each shared the fear of dying alone without relatives. Furthermore, the picture of the burial team lingered in participants’ minds as they felt they too were heading toward the same fate of dying. These fears arose from witnessing patients dying while receiving care. One participant described her fear of dying as feeling like a man with a gun was following her and waiting to pull the trigger. Participant 2 explained the cause of her worry.

Of course, when you learn that the disease is incurable and it’s Ebola, and you will die. I also got worried because there was no way out because the moment you contract the disease, and you will die, so I was also worried.

Participant 1 was also worried and offered,

According to the experience of what I saw while attending to some of my colleagues, you may be there for one or two days, but after that, when those symptoms of Ebola appear, you know you are automatically going to die. So, after getting the disease, my feeling was that I am going to die. The picture I would draw is that of the burial team, which remained in my sight. Otherwise, I was a candidate to go.

### Feeling Hopeless Yet Hopeful in God’s Mercy

The data revealed that participants felt hopeless based on their knowledge that the disease is fatal. Even so, they still hoped God would spare them. In those times, they resorted to self-counseling about their physical condition, while anticipating survival depending on the individual health condition. Furthermore, they were counting down to the days. The death of colleagues made some participants meander between survival and death. Although some were frightened by the unfolding painful events, others hoped and prayed it was not their time to die. Their prayers were for survival, protection of caregivers, and for the ability to repent believing that if one is to die, their relationship with God should be worthy and holy.

Participant 2 offered,

I had surrendered it (my life) for death now, but through His miracle, things began to stabilize, it wasn’t easy.

Participant 5 also recalled feelings of dying and explained,

for me, I had lost hope. My feeling was that I am going to die. At the same time, I said Lord, if it’s my time, I am going to go.

And Participant 1 recounted,

I counsel myself, and sometimes I say I might die, but sometimes you might get frightened, sometimes you feel are losing your breath. You feel you are more ill, but from there you have to get courage. For me, I had hope because I believed, I said God; it’s not my time!

Participant 5 had similar musings and stated,

I thought I was going to die, so when I survived, I said I think there is something the Lord still wants me to do; that’s why he made me survive but many have been infected with this Ebola, and they died. So, I was even questioning myself; why? So, the only answer that I give to myself I think there is something the Lord still wants me to do otherwise I could not have existed up to today.

### Longing to Connect With Others

This theme emerged from participants’ feelings of not being encouraged or talked to courteously by fellow nurses caring for them in isolation units. Participants felt that when colleagues delivered food wearing gloves, they were showing fear. Participant 2 recalled,

When you are sick, and someone comes to give you bread, even putting on gloves when someone fears you excessively. You get it ehh! You really feel so bad and being isolated, yet we wanted people to see us, but no one was able to visit us.

Furthermore, isolation and lack of company, except for health care workers and some religious leaders, led to loneliness. During these times, while they were alone in the isolation room, participants devised measures to counter internally generated fear and counseled themselves. This self-counsel for participants was a way of preparing for death, to which they had already surrendered. Participants admitted that the death of staff and other patients due to EVD also aggravated the situation.

### Suffering Health Disorders

Participants described their experience of suffering from physical and mental disorders. Physical disorders occurred in the acute phase but continued. Short-term bodily ailments were bleeding from orifices. The medium-term bodily ailments felt were pain, blurred vision, loss of libido, and general body weariness. The most significant long-term ailment experienced by some was pain particularly, chest pain, backaches, and occasionally palpitations. Participant 4 offered,

Pain is my major problem. I tried to move in the hospital to check that everything is normal. Sometimes, I have palpitation and chest pain. Sometimes, when it is severe, I go to the hospital, and they put me on propranolol and paracetamol.

Another, Participant 3 stated,

When I was discharged, I was not even feeling very well. I was going to Lacor every two weeks for check-up. For almost two years we were going to get treatment; we were feeling dizzy, I was very sick and weak, and I could not walk properly.

Mental health disorders experienced by the surviving nurses included symptoms of posttraumatic stress as they recall the trauma of the experience. The lingering trauma was evident in their body language that showed misery, grief, sorrow, worry, and even guilt. During the interviews, Participant 5 broke down in tears before narrating how she suffered and recovered from EVD:

When I see the people whom I was with, when I go there and see the people, and when I am in the place, if I just remember, I say it was a bad tragedy. I do recall it, and when you remind me, I don’t always like to be reminded about it. When you remind me, I get stressed and think of my colleagues who passed on.

For two participants, the misery was further compounded by their physical body image and the disease states such as edema, anemia, and swollen abdomen. Furthermore, the reverberating sounds of individuals weeping over the loss of a colleague, friend, or relative also aggravated the misery and guilt. For Participant 2,

Being a survivor is not far from people who died of it. Those who died of Ebola, the only difference is that their people missed them forever, but I think even people who survived it have a lot of issues ehh!

### Being Ostracized

Faced with stigma and rejection, participants felt they were being ostracized. Participants experienced stigma in various forms. They suffered psychological torture from family members. They were further isolated as they experienced the community burning of clothes, refusal to eat with survivors, avoidance of a handshake when greeting survivors. Similar forms of being ostracized targeted their children. For example, they were prohibited from fetching water and even playing football in the community. For some, they also felt rejected by family, neighbors, friends, and professional colleagues who labeled them “Ebola nurses.” A few participants lost friends and felt they were alone. Participant 2 stated,

People first feared to come near me, even my children they never wanted them to play with their children. They prevented my children from fetching water. After I was discharged from the hospital, people abandoned and feared me, even my relatives and neighbours. The community feared they would contract Ebola.

Another, Participant 3, described the situation as

I have lost friends. I was just there lonely and am just to be by myself like that. I was by myself, and people come and go. There is no time to interact with them or to share other things that we used to.

### Feeling Neglected and Betrayed by Organizational Systems

The lack of disclosure surrounding the status of the disease caused participants to live in uncertainty. They recounted that initial positive test results were not shared with them immediately. This approach of limited or nondisclosure left many unanswered questions. Some only realized they had Ebola when they were admitted to another hospital and placed in isolation. Participants thought they were being transferred to eliminate the possible stigma from other nurses who might fear caring for them. Participant 1 explained,

When they took me to the hospital, by then those people, the doctors from South Africa they had come, then they tested my blood straight away, they found that I was a victim of Ebola, but they didn’t tell me.

Participants claimed a team from the CDC took their blood samples. Participant 4 explained, “our blood was being taken without any explanation.”

Participants also felt they lacked adequate protective equipment to protect themselves at the beginning of the outbreak. Lack of protective gear compromised quality of care provided to patients and the affected nurses themselves. The practice was that gloves were to be worn for specific procedures, yet when patients came in with a fever, and malaria-like symptoms, nurses did not use gloves. Participant 5 in describing the situation on the wards acknowledged that “[they] had no gloves, and [they] were using bare hands. Little did [they] know there was a deadly disease.”

Participants also shared that they felt neglected and were treated differently from doctors who were infected. Furthermore, families of doctors received compensation, and their children were supported. Participants claimed they were not recognized for their efforts, risking their lives to care for EVD patients. One participant maintained that the only time she got any recognition was at the international nurse’s celebration. There, she was publicly recognized for her bravery. Interestingly, despite the challenges they faced some participants maintain they would still consider providing nursing care to Ebola patients should another outbreak occur. Others felt they would find it too difficult.

## Discussion

The study revealed the meanings the five nurses gave to their life during and after Ebola. The six themes that emerged from the data are: living in fear of dying, feeling hopeless yet hopeful in God’s mercy, and longing to connect with others, suffering health disorders, being ostracized, and feeling neglected and betrayed by organizational systems. The findings herein document the voices of the nurses who survived Ebola and records their selfless dedication to saving others. Interestingly, the experiences of these nurse survivors’ receiving care while in isolation units were inconsistent with the ideal nursing care provision. On a scale, they measured their treatment as ranging from being good to poor.

The themes of living in fear of dying and feeling hopelessness dominated the expressions of the survivors. The analogy of fear as a gunman waiting to pull a trigger affirms the findings of other studies. For example, Locsin et al. (2009) used artistic expression to describe the unique meaning of living a life while consciously waiting to know, while giving one’s life so that others may live. Similarly, Kongsuwan et al. (2016) alluded that nurses in emergency rooms had fears of dying.

The health disorders encountered included, bleeding from orifices, pain, blurred vision, loss of libido, and general body weaknesses. Some ailments still exist 14 years postevent (Jacobs et al., 2016). This finding is consistent with Simeone et al. (2015). These authors suggest that while providing care to survivors from a clinical perspective, providers also need to deliver more psychosocial interventions to help survivors. These interactions will enable surviving nurses to cope with life’s changes and encourage them to adapt to the daily limitations caused by an illness. However, if the care provided to people with critical illnesses does not become a “shared mutual process,” it results in delayed healing for the victim. Delayed healing is likely to have clinical implications for survivors and may consequently affect their nursing practice.

The finding of being ostracized was consistent with Locsin and Matua’s (2002) and Matua’s (2014) deliberations. These authors argued that victims of Ebola were ostracized, rejected and traumatized. In a similar vein, Bell et al. (2017) described finding from their study of 58 participants from a variety of health care workers including doctors, nurses, community health care workers, and traditional birth attendants. All experienced stigmatization following the EVD outbreak.

The way nurses vividly recalled and narrated their ordeal suggested they still had strong emotions tied to the experience. Although, they consented to participate in the study, clearly recollecting their experience triggered sad memories. Some survivors were very emotional and admitted they rarely talk about EVD. Similarly, Storli et al. (2008) asserted that strong emotions do exist in the lives of survivors. Furthermore, Locsin et al. (2009) noted that some experiences are so embedded in a person’s life that it becomes difficult to forget.

The need to be connected to others had a deep impact on participants’ lifestyles. The isolation altered their social interactions with family, relatives, and friends, which caused them to turn to God. This finding is similar to previous studies in situations of hopelessness and helplessness, where individuals turned to God for guidance and strength. Storli et al. (2008) affirmed when survivors feel supported by family members and others, they will respond by sharing, which strengthens survivors to go on to overcome the effects of the traumatic event (Blevins et al., 2019; Raven et al., 2018).

The theme of being betrayed and neglected by the organizational system was due to challenges such as lack of knowledge of infectious diseases, poor infection control, and lack of care procedures before an Ebola outbreak. The absence of PPE, such as masks and gloves, endangered the lives of nurses. This finding is similar to the findings of another study conducted in Africa in which participants, healthcare workers from Norway, felt neglected while putting their lives at risk to save others (Andertun et al., 2017). The narratives provided unequivocal evidence of the commitment these nurses had to provide nursing care to their patients. Despite the risks to their own lives, lack of social, intuitional and government support, they exemplify the essence of nursing. In the end, their experiences gave them an appreciation of the other (the person dying of EVD), and they embodied an authentic expression of best in nursing (Hayter, 2015). Thus, embodying an authentic expression of understanding self and others.

## Conclusion and Implications for Policy, Education, and Research

This article offers an understanding of the experiences of five Ugandan nurses who contracted and survived Ebola while caring for patients with the illness. During their ordeal, these nurses were aware that their survival meant living in fear of dying. As well, they were in constant fear of the unknown while feeling ostracized and rejected by others. Furthermore, they endured bodily aches and pain, psychological trauma and resulting posttraumatic stress disorders. These serve as daily reminders of the personal war they endured while being infected with Ebola.

Results demonstrate the urgent need for evidence-based interprofessional education and training. These education programs should be developed in collaboration with nurses who survived as their personal experience would add the front line perspective. Not only should they include the voices of nurses that survived EVD, but they should include other health care professionals, community partners, governmental agencies, and other stakeholders, which will allow for improved knowledge about EVD and other potential infectious diseases that will occur, for the threat is imminent. Furthermore, it would help health care professionals to devise and implement policies and frameworks to protect themselves and others, while providing immediate care and information about where to seek additional resources. With the emergence of the COVID-19 and its variant strain, these findings go along way to inform health care workers and authorities of the glaring need for proper information channels and use of PPE gears to keep nurses and patients safe. COVID-19. With proper information and the right gears, the lives of many nurses could be saved from the Ebola epidemic. The same could be true for those now experiencing COVID-19.

Nursing plays an important role in healthcare and society; therefore, nurses must be engaged in all levels of the policy-making process to strengthen current health care systems. Nurses should join forces with their professional organizations including the International Council of Nurses, and the Ugandan Nurses and Midwife Council, to advocate for and give voice to the essential contribution nursing can make in building a safety net of compassionate health care for all Ugandans.
